# The relationship between hotel star rating and website information quality based on visual presentation

**DOI:** 10.1371/journal.pone.0290629

**Published:** 2023-11-02

**Authors:** Ching-Hsue Cheng, Ming-Chi Tsai, Yuan-Shao Chang

**Affiliations:** 1 Department of Information Management, National Yunlin University of Science and Technology, Douliou, Yunlin, Taiwan; 2 Department of Business Administration, I-Shou University, Kaohsiung City, Taiwan; TU Wien: Technische Universitat Wien, AUSTRIA

## Abstract

The hotel industry is essential for tourism. With the rapid expansion of the internet, consumers only search for their desired keywords on the website when they trying to find a hotel to stay, causing the relevant hotel information would appear. To quickly respond to the changing market and consumer habits, each hotel must focus on its website information and information quality. This study proposes a novel methodology that uses rough set theory (RST), principal component analysis, t-Distributed Stochastic Neighbor Embedding (t-SNE), and attribute performance visualization to explore the relationship between hotel star ratings and hotel website information quality. The collected data are based on the star-rated hotels of the Taiwanstay website, and the checklists of hotel website services are used to obtain the relevant attributes data. The results show that there are significant differences in information quality between hotels below two stars and those above four stars. The information quality provided by the higher star hotels was more detailed than that offered by low-star hotels. Based on the attribute performance matrix, the one-star and two-star hotels have advantage attributes in their landscape, reply time, restaurant information, social media, and compensation. Furthermore, the three-five star hotels have advantage attributes in their operational support, compensation, restaurant information, traffic information, and room information. These results could be provided to the stakeholders as a reference.

## 1. Introduction

According to [1], the number of visitors to Taiwan in 2016 years exceeded 10.69 million. It grew to 10.73 million in 2017. In this era of rapid change, tourism has become a global consumer market that cannot be ignored, especially in tourism consumption patterns and technological development [2]. Furthermore, understanding consumer preferences and needs and taking advantage of marketing have become essential to the success of the tourism industry.

In this era of internet expansion and rapid information growth, as long as a person searches on the internet, he or she can easily find the necessary information. Travelers, for example, search for information on the internet to determine their travel itineraries and accommodations [3]. Therefore, many hoteliers utilize information technology to develop websites for effective marketing and to attract travelers. These websites cater to the diverse needs of visitors and attract the attention of visitors by the quality of the information presented on the website. Internet marketing is not constrained by space or time [4]. Travelers from non-local areas or foreign countries who have never visited their intended destination do not know the cultural conditions of the place. Therefore, the information provided by the hotel website is important for determining travel information to reduce this information gap. In this way, website information can assist a traveler as the basis for decision-making [5].

Service quality is an abstract concept whose factors are not easy to describe [6, 7]. Service quality also has various characteristics, such as intangibility, heterogeneity, and inseparability [8–10]. To maintain online service quality, Li, Tan, and Xie [11] suggested that the information provided by a website should be able to meet the customer’s requirements to help them solve problems. Moreover, Aladwani and Palvia [12] proposed the concept of E-Service Quality (e-SQ), noting that excellent service quality should include the following attributes: proximity, easy navigation, efficiency, flexibility, reliability, personalization, security/privacy, responses, guarantee/trust, aesthetics, and price information.

Tourism in the global consumer market is no longer negligible. Customers are often tired after traveling all day and hope to find high quality and comfortable hotels to stay. Previous hotel research used statistical methods or questionnaires [6, 7, 9, 10]. However, to our knowledge, data mining has not been used to study hotel-related issues, and the sample sizes were relatively small in related studies. Therefore, this study is the first to apply data mining and visualization tools to expand the sample size and analyze the relationship between star-rated hotels and their website information quality. Based on the above mentioned, this study proposed a novel methodology to explore the relationship between hotel star ratings and website information quality. The contributions of this study are as follows:

- Based on practical application, this study examines previous studies to find the attributes of website information quality for star-rated hotels and collect the number of samples to increase the usefulness of the study;
- Website quality is a multidimensional aspect that affects consumer attitudes and induces satisfaction, which includes many attributes. The key attributes that influence the star-rated hotel can allow consumers to differentiate between different competitors. Therefore, this study uses rough set theory to find the key attributes of website information quality for star-rated hotels;
- Previous studies most used statistical analysis methods to research website quality and star-rate hotels, and no rules are generated. Therefore, we apply rough set theory to generate the rules for the website information quality of the star-rated hotels for consumers to identify star-rated hotels; and
- For the visual representation, we use t-Distributed Stochastic Neighbor Embedding (t-SNE) and the principal component analysis (PCA) visualization tool to determine the number of classes and attributes. Further, this study applies an Alternating Least Squares sCALing (ALSCAL) algorithm to show the attribute performance matrix on the axis of information quality and importance.

The remaining content of this paper is organized as follows: Section 2 features the related work, including the information quality of the hotels, star-rated hotels, attribute selection, rough set theory, and classification techniques. Section 3 describes the concept and the proposed procedure. Section 4 illustrates the collected datasets, experiments, comparisons, and findings; the final section is the conclusion.

## 2. Related work

This section introduces the related literature, including the website information quality of the hotels, star-rated hotels, and classification technology.

### 2.1 Website information quality

Taiwan’s tourism and accommodation industry have been promoted by government policies, backpackers, and free travel, which has led to a significant increase in the number of tourists coming to Taiwan, especially from the new southbound countries. From 2014 to 2017, the number of hoteliers and rooms grew significantly, but there was still a shortage during the hot season.

Since the late 1990s, the Internet has revolutionized business operations in the hotel industry, and the internet has become a powerful operational and marketing tool [2, 13]. MacKay and Vogt [14] noted that information technology was established as a necessary factor for continuous innovation. A quality website is profitable, easy to use, accessible, useful, and offers reliable information [3, 4]. Such a website must have a good design and visual appearance and meet user needs and expectations [15]. For programmers, website quality refers to maintainability, security, and functionality. End-users pay more attention to usability, efficiency, and creditability. Website quality is the main factor in e-commerce because customers care about the quality of a website, which directly influences their purchasing intentions [16–18].

Many travelers use the internet to search for pictures of rooms, facilities, and other services. Travelers also look at reviews for a hotel before deciding whether to book the hotel. Sparks and Browning [19] investigated the impact of online reviews on hotel booking intentions and consumer trust. The results showed that consumers might be affected by negative information.

### 2.2 Hotel star rating

At present, the international classification of hotels uses a five-star rating system: One star is the lowest rating, and five stars are the highest. The appearance of the building, the interior decoration, the equipment and facilities, and the management system of a hotel are usually used as the star rating criteria. Therefore, hotels with a higher star rating are more luxurious than hotels with a lower star rating. Moreover, the higher star rating hotels have better service quality. However, different countries have different national situations and cultures, and these star ratings are still based on individual national criteria. Guillet and Law [20] explained that the hotel level was determined by the hotel’s quality and ratings. These hotel ratings are based on laws approved by the state or local governments or based on the evaluation criteria established by national tourist organizations, hotel associations, travel consumer organizations, guidebooks, travel websites, and volunteer organizations.

Fang, Ye, Kucukusta, and Law [21] also noted that the overall quality of a hotel could be inferred from the star ratings provided by impartial official organizations. Consumers most commonly use star ratings to choose a hotel [22]. Furthermore, the star-rated system is the most frequent customer segmentation model in the hospitality industry [23]. Star ratings represent the quality of all services provided by the hotel and its market position, which will help to improve the overall service level of the hotel. At the same time, they segment the marketing and provide the basis for consumers with different needs to choose a hotel; stars also serve as a reference to improve the system of star-rated hotels according to the Taiwan tourism bureau.

The attributes of the determined hotel ratings include customer satisfaction, price, hotel environment, cleanliness, service, attractiveness, opportunity to relax, loyalty program, guest experience, promotions, facilities, meetings, green programs, hotel image, hotel reputation, and many others [24–29]. Shanka and Taylor [30] analyzed the 18 services and facilities factors into three dimensions: physical facilities, room facilities, and reception services. Of these three dimensions, guests of Australian three-star hotels regarded experienced reception as the most important.

### 2.3 Classification technique

Rough set theory is mainly used to find the core set and generate classification rules in this study. Related classification techniques were also applied for comparison with the proposed procedure. These methods are introduced in the following.

#### (1) Rough Sets Theory

Rough Set Theory (RST) was proposed by [31] and was mainly used to process the ambiguity and inaccuracy of knowledge. It is applied in data mining. Pawlak [32] noted that RST had strong qualitative analysis capabilities, which did not require properties or assumptions in advance. Rather, RST can generate the internal rules of the data directly from the attribute set of the data. RST is a non-parametric method that has its foundation in traditional set theory and has been widely used in decision problems [33]. Furthermore, RST can explore the relationship between data attributes and has been applied in many fields, such as decision analysis, knowledge discovery, expert systems, and pattern recognition. The advantages of RST [32] are described as follows. (1) No preliminary or other information about the data is required. (2) It provides effective methods, algorithms, and tools to find hidden patterns in data. (3) It reduces the attributes and data dimension. (4) It evaluates the importance of attributes. (5) It allows the automatic generation of decision rule sets from the data. (6) It provides a direct explanation of the results obtained. (7) It allows parallel distributed processing. Furthermore, RST has been widely used in academic research fields such as artificial intelligence, medical diagnostics, pharmaceutical research, program control, credit fraud detection, bankruptcy detection, the stock market, marketing research, climate change, and expert systems [34].

#### (2) Decision tree C4.5

The decision tree algorithm used in this study is a C4.5 learning method proposed by [35] and extended by Quinlan’s ID3 (Iterative Dichotomiser 3) algorithm [36]. Decision tree analysis technology can be used simultaneously for continuous variables and categorical variables and can be applied to the analysis of predictive classification but cannot be used for time series [37, 38]. Lin [39] applied a decision tree based on a technical target to predict the daily up and down of stock prices to promote return on investment. Nasseri, Tucker, and de Cesare [40] combined a decision tree with text mining to analyze the semantic web and explored whether the information on the social network affects changes in the stock price. Hastie et al. [41] noted that the most widely used decision tree algorithms were CART (Classification and regression trees), CHAID (Chi-square automatic interaction detector), ID3, C4.5, and C5.0.

#### (3) Random forest

Breiman [42] registered the name of random forest. Random forest is a kind of integrated multi-decision tree for classification and regression prediction [42–44] and offers a method for combining multiple random trees (forest) into one big classifier with greater randomization. Thus, the random forest is an ensemble method usually applied to random trees. Kampichler et al. [45] conducted a comparison of five algorithms (decision tree, random forest, artificial neural network, support vector machine, and fuzzy model). Their results showed that the random forest and decision tree offered better performance than the other algorithms. Hsieh [46] also applied the random forest, support vector machine, and neural network methods to construct clinical decision support tools for the diagnosis of acute appendicitis and breast cancer. Their results showed that random forest provided better performance.

#### (4) Random tree

Aldous [47] first introduced the random tree. It is an integrated learning algorithm with a supervised classifier, which can generate a large number of individual learners. The random tree uses the idea of bagging to construct a set of random data for building a decision tree. In the standard tree, each node is split according to the best split between all variables [48]. The random tree is essentially a combination of two existing algorithms in machine learning: a single model tree combined with the idea of a random forest. The model tree is a decision tree in which each leaf has a linear model that is optimized for the local subspace interpreted by the leaf. The random tree is already proven to improve the performance of a single decision tree significantly. The diversity of trees is created by two randomization methods [42, 49].

#### (5) Support Vector Machine

Support Vector Machine (SVM) is the latest emerging classification method but can be traced back to 1963 [50]. SVM was formally proposed by Vapnik of Bell Labs in 1995. As a method of machine learning, this method is widely used in statistical classification and regression analysis. SVM is based on statistical learning theory, the principle of risk minimization, and the effective separation of different types of data. It has been successfully applied to the topic of classification [51–54]. This study used the LibSVM program of SVM, which was developed by Chang and Lin [55].

## 3. Proposed method

Recently, the Taiwanese government has actively broadened the tourism market with self-guided trips (backpacking travel), European and American long-distance airlines, new southbound countries. The number of tourists not only depends on Chinese tourists coming to Taiwan but also tourists from other countries who visit Taiwan. According to the data of the Taiwan Tourism Bureau, the number of tourists from new southbound countries has significantly increased. The number of domestic tourists in 2017 was 183.45 million, which was the second-highest amount in the past six years, second only to the amount in 2016. In the hotel industry, the number of hotels and rooms increased from 8,232 hotels and 176,519 rooms in 2014 to 11,518 hotels and 221,818 rooms in 2018. There was, therefore, obvious growth in the hotel industry with the phenomenon of insufficient supply.

Past studies of hotel information quality had some shortcomings: (1) Taiwan hotel databases were not very complete. (2) Some studies only used small samples, thereby producing different results. (3) There was relatively little research on hotel website information quality. (4) The different research methods had different biases. (5) The service quality and evaluation of hotel star ratings almost used statistical methods or questionnaires. Data mining and visualization tools can improve the shortcomings (2), (4), and (5) of past studies of hotel information quality. Because data mining and visualization technology can show data results in a way that is easy for humans to understand, and this study collects data from websites that can expand the research sample size, and the two tools can present the relationship between star-rated hotels and their websites information quality. In this study, data mining techniques include five attribute selection methods and five classifiers. Attribute selection can select the important attributes to reduce the dimensions of the dataset and improve the performance of the data-mining algorithm. Classifiers can identify the pattern of new samples and generate classification rules.

The visualization tools include PCA [56–59] and t-SNE [60] to visualize the class data for determining the number of classes. ALSCAL [61] was also applied to build an attribute performance matrix for segmenting the attributes of the different star-rated classes. PCA is a dimensionality reduction technique commonly used in data analysis and visualization. PCA can be plotted with the first and second principal components as a preprocessing step to facilitate visualization. Here are some reasons for using PCA in this study: (1) Dimensionality reduction: PCA helps reduce the number of variables in a dataset while preserving essential information. PCA simplifies the visualization process by transforming high-dimensional data into a low-dimensional space [62]. (2) Visualization of high-dimensional data: PCA allows data to be projected into a low-dimensional space, usually 2D or 3D, which can be easily plotted on a graph [62]. This facilitates the exploration and interpretation of complex datasets. (3) Identify patterns and clusters: PCA can reveal underlying patterns, trends, and relationships by visualizing the principal components in data. (4) Attribute importance and contribution: PCA provides information about the importance and contribution of the original variables to the principal components. t-SNE is a nonlinear visual dimensionality reduction approach using deep learning. t-SNE is easy to optimize the low-dimensional manifold structures, which is a nonlinear embedding method used in several research areas. ALSCAL algorithm is a multidimensional analysis (MDS) procedure, which can analyze any type of two- or three-way data (the nominal, ordinal, interval, or ratio data). MDS considers the different dimensions of relationships and aims to explore data and search for potential or hidden connections between dimensions.

**Fig 1:**
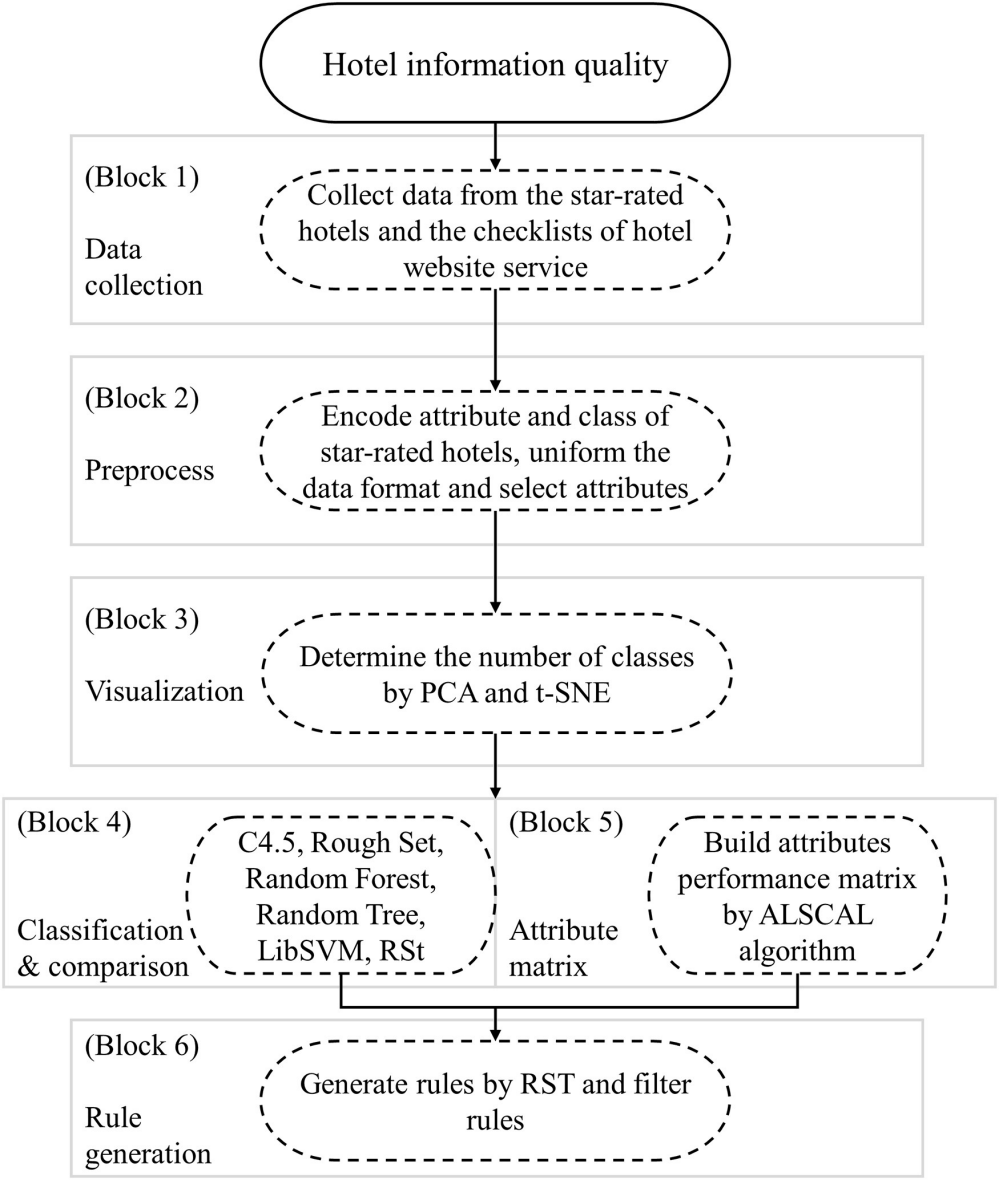
Procedure of proposed method.

Based on the five main facets above, this study proposed a computational procedure for the proposed method that included six steps (blocks), as shown in Fig 1. The detailed computational procedure includes five steps: data collection, preprocessing, determining the number of classes by visualization, classification, and evaluation. Each step is described in detail as follows.

### Step 1: Data collection

In Taiwan, there were more than 7,000 hotels in 2019. Each year Taiwan Tourism Bureau will evaluate each hotel’s star rating, from one star to five stars. This present study only collected the data of star-rated hotels taken from the Taiwanstay website of the Taiwan Tourism Bureau [63], with 453 star-rated hotels in 2019. This study used the names of 453 star-rated hotels to collect their website information. However, five hotel names were duplicated, 19 hotel websites had moved or could not be found, and one hotel was out of business. Because some hotels were renamed, they did not match the original names on the list. Accordingly, this study updated each original name to its new name. After confirming the 428 star-rated hotels, we searched the websites of the 428 hotels to collect data according to the checklists of hotel website services, as in Table 1 [64]. The final, collected dataset included the website data for 428 hotels from one star to five stars, and all 23 attributes were divided into 12 dimensions.

**Table 1:** Collected dimension and attribute.

Dimension	Attribute	Abbreviation
Completeness	1.Room information	1. RoomInfo
	2.Restaurant information	2.RestaurantInfo
	3.Conference/banquet hall information	3.ConferenceHall
	4.Information of other facilities	4.OthFacInfo
	5.Traffic information	5.TrafficInfo
Relevance	1.Surrounding sights	1.Landscape
	2.Preferential services	2.Compensation
	3.Outward linkage	3.Contact
Timeliness	Information update	InfoUpdate
Esthetics	Interface design	InterDesign
Ease of use	1.Operational supports	1.OperaSupport
	2.Multi-language designs	2.Language
Navigability	1.Page-touring function	1.Navigation
	2.Video and virtual reality	2.VR
Interactivity	1.Interactive services	1.Interaction
	2.Information exchange	2.MessageChange
Virtual involvement	Social networking	SocialMedia
Assurance	1.Hotel Corporate Image System	1.HotelImage
	2.Authentication and cooperative symbols	2.Authenticate
Security	Secure certification	Security
Responsiveness	Response time	ReplyTime
Empathy	1.Complementary information	1.OtherInfo
	2.Potential services	2.PotentialService

### Step 2: Preprocessing

There are three sub-steps in this step.

1. Encode attribute values: The collected data are categories; hence, this study encoded the “Y” and “N” into 0 and 1.
2. Handle imbalanced classes: Because the number of different star hotels was not the same (as shown in Table 2), to handle the imbalanced classes, this study merged the different star-rated hotels into three classes and two classes, as shown in Table 2. In three classes, we combined the one-star and two-star hotels into class A, the three-star hotels into B, and the four-star and five-star hotels into class C. In two classes, we combined the one-star and two-star hotels into class A and the three-star, four-star, and five-star hotels into class B.
3. Select attributes: To reduce the dimensions of the dataset and obtain the important attributes [65], this step applied five attribute selection methods: Correlation-based feature selection (Cfs) [66, 67], Correlation [68, 69], InfoGain [70, 71], ReliefF [72, 73], and GainRatio [74, 75].

**Table 2:** Classes of merged the different star-rated hotels.

Hotel level	Three classes	Two classes
Star 1 (6 hotels)	A (121 hotels)	A (121 hotels)
Star 2 (115 hotels)		
Star 3 (196 hotels)	B (196 hotels)	B (307 hotels)
Star 4 (46 hotels)	C (111 hotels)	
Star 5 (65 hotels)		

### Step 3: Determine the number of classes by data visualization

Information visualization means that after a large amount of data is processed, the information is represented using human-friendly graphics [76]. A successful visualization of a concept can help users quickly grasp and absorb the expressed information [77]. This step applied the PCA component scores 1 versus the PCA component 2 scores to draw a scatter plot of different classes and utilized t-SNE visualization tools to depict the data distribution of different classes. The t-SNE is better than PCA; it can allow high-dimensional data to be imaged into low-dimensional spaces, making each point on the data clearer via visual scatter diagrams.

### Step 4: Classification and comparison

To explore the relationship between hotel star rating and hotel information quality, this step applied a rough set classifier to classify the collected dataset and compare its accuracy with five other classifiers, including C4.5 [35], Rough Set [31], Random Forest [42], Random Tree [49] and LibSVM [55]. This step used ten-fold cross-validation for the experiment and applied classification accuracy to evaluate the comparisons.

### Step 5: Build the attributes performance matrix by ALSCAL

This step used the ALSCAL algorithm to discriminate the weak and advantageous attributes for different star-rated hotel classes. After executing ALSCAL algorithm, attribute performance visualization provided the weak and advantageous attributes of the different star-rated classes, and the attribute performance matrix shows what attributes need to be enhanced for different star-rated hotel classes.

### Step 6: Rule generation

Based on step 4, the best classifier offers optimal accuracy. Thus, the best classifier generates the rules of the star-rated hotel. When representing the rules, the generated rules are filtered. *i*.*e*., each rule must match at least two data points; otherwise, we delete the rule.

## 4. Experiments and results

This section provides the collected data, experiments, and comparisons to find the relationship between hotel star ratings and hotel information quality. Based on the analyzed results, the competitive advantage of the hotel is also explored [78–80].

### 4.1 Star-rated hotels dataset

The data source is a list of 453 star-rated hotels in Taiwan provided by the Taiwan Tourism Bureau. This study used a simple crawler program to capture the information quality of the 453 hotel websites according to the checklists of the hotel website services, as shown in Table 1. Then we checked for the presence of the matching attributes from Table 1, such as room information, conference information, other equipment information, etc.

In practice, the collected star-rated hotel data were based on the list of star-rated hotels from the Taiwanstay website of the Taiwan Tourism Bureau [63]. There were a total of 453 star-rated hotels in 2019. Among the 453 star-rated hotels, five hotel names were duplicates, 19 hotel websites had moved out or could not be found, and one hotel was out of business. Some star-rated hotels had been renamed. Hence, we updated their name to the new name, the collected dataset included the website data of 428 hotels from one star to five stars, and the 23 attributes were divided into 12 dimensions.

### 4.2 Experiments and comparisons

This section is based on the proposed computational procedure to execute the experiments and comparisons. Before conducting the experiments, we applied Pearson’s correlation to explore the relationship between star-rated hotels and the factors of information quality. We used a one-way ANOVA to test the differences between different star-rated hotel classes. All analyses are represented in the following.

#### A. Relationship between star-ratings and information quality factors

This study used 23 attributes and star levels (from one star to five stars) to calculate Pearson’s correlation coefficient and the correlation between the 15 attributes and star levels is significant at a 0.01 level. The correlation of the three attributes is significant at a 0.05 level, but the traffic information and landscape attributes do not have a significant correlation between star levels. Moreover, the collected dataset provided a value of 1 for all the room, navigation, and interaction attributes. This means that all 428 star-rated hotel websites have three information quality elements. Based on Pearson’s correlation equation, the three attribute values are constant (all attribute values are the same) and are not calculate Pearson’s correlation. Ultimately, there are 18 attributes related to star levels.

#### B. Attribute selection

This part used five attribute selection methods to determine the important attributes and rank the ordering of important attributes for two and three classes. Table 3 shows the results of the two classes. In the top 10 rankings, we found that 10 attributes appeared at least three times, as shown in the InfoGain and ReliefF columns of Table 3. Based on the Condorcet ranking method [81], the ordering of the important attributes is Conference Hall, OthFacInfo, Language, Compensation, Restaurant info., Social media, Reply time, Info update, VR, and Potential service. Thus, the website information of star-rated hotels should consider these important attributes to meet their customers’ needs, especially international hotels. Many enterprises rent the conference halls of star-rated hotels as a location for their activities or meetings, but the other facility information is the customer’s concern. In addition, each star-rated hotel should disclose its existing room information and equipment information, such as its swimming pools, playrooms, and business centers. Potential service is also a consideration for customers when browsing a hotel website, such as whether they offer a washing machine or dryer, bicycle rentals, or wake-up calls.

**Table 3:** Results of attribute selection for two classes.

Rank	InfoGain	ReliefF
1	Conference Hall	ConferenceHall
2	OthFacInfo	OthFacInfo
3	Language	Language
4	Compensa.	SocialMedia
5	Resturant	ReplyTime
6	SocialMedia	Opera Support
7	ReplyTime	Compensa.
8	InfoUpdate	Resturant
9	Potential Service	Potential Service
10	VR	InfoUpdate
Accuracy	86.44	86.44

For three classes, eight attributes from the five attribute selection methods appear at least three times in the top 10, based on the Condorcet ranking method [81] used to rank the ordering. The key attributes are conference hall, multi-language, other facility information, compensation, social media, restaurant information, reply time, and virtual reality (VR); the eight attributes are the same as the key attributes of the two classes. This means that for both high and low star-rated hotels, the selected key attributes are essential for the hotel websites and that all customers are most concerned about the selected key attributes. As a result, this information should be fully presented on websites. Hence, the customers can more conveniently understand the relevant hotel information, thereby increasing their willingness to book when browsing the hotel website.

#### C. Differences between the star-rated hotels and their information quality

To understand the differences between the star-rated hotel and the selected attributes of information quality, this study used an ANOVA analysis to test the difference between the three classes and the selected eight attributes. The test results of three classes (A<B<C) show that hotels with higher star ratings are better than hotels with lower star ratings in terms of the important information quality of their websites. The Scheffe comparison of the selected eight attributes reveals a significant difference; only VR has no significant difference of A<B. This means that the four-star and five-star hotels have VR facilities, but the one-, two-, and three-star hotels do not have VR facilities. A t-test was used to test the differences between the two classes and the selected 10 attributes. The test results of the two classes show that A<B has a significant difference, and it means that hotels with higher star ratings are better than hotels with lower star ratings in terms of the selected information quality of their websites.

#### D. Determining the number of classes by data visualization

**Fig 2:**
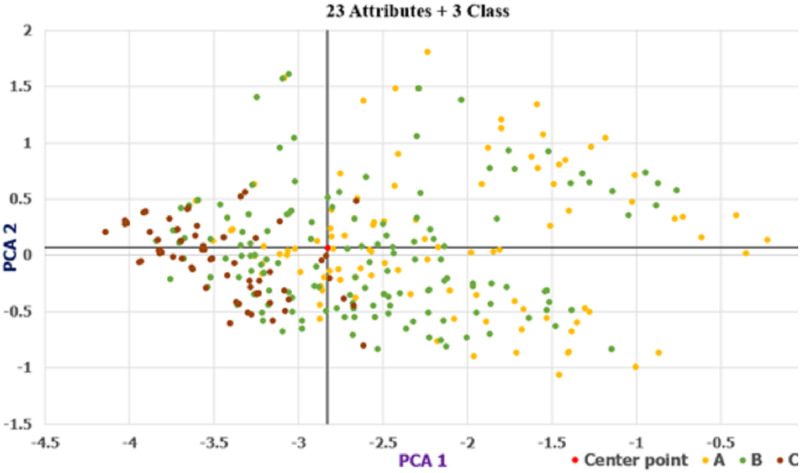
Scatter of PCA for three classes (23 attributes).

**Fig 3:**
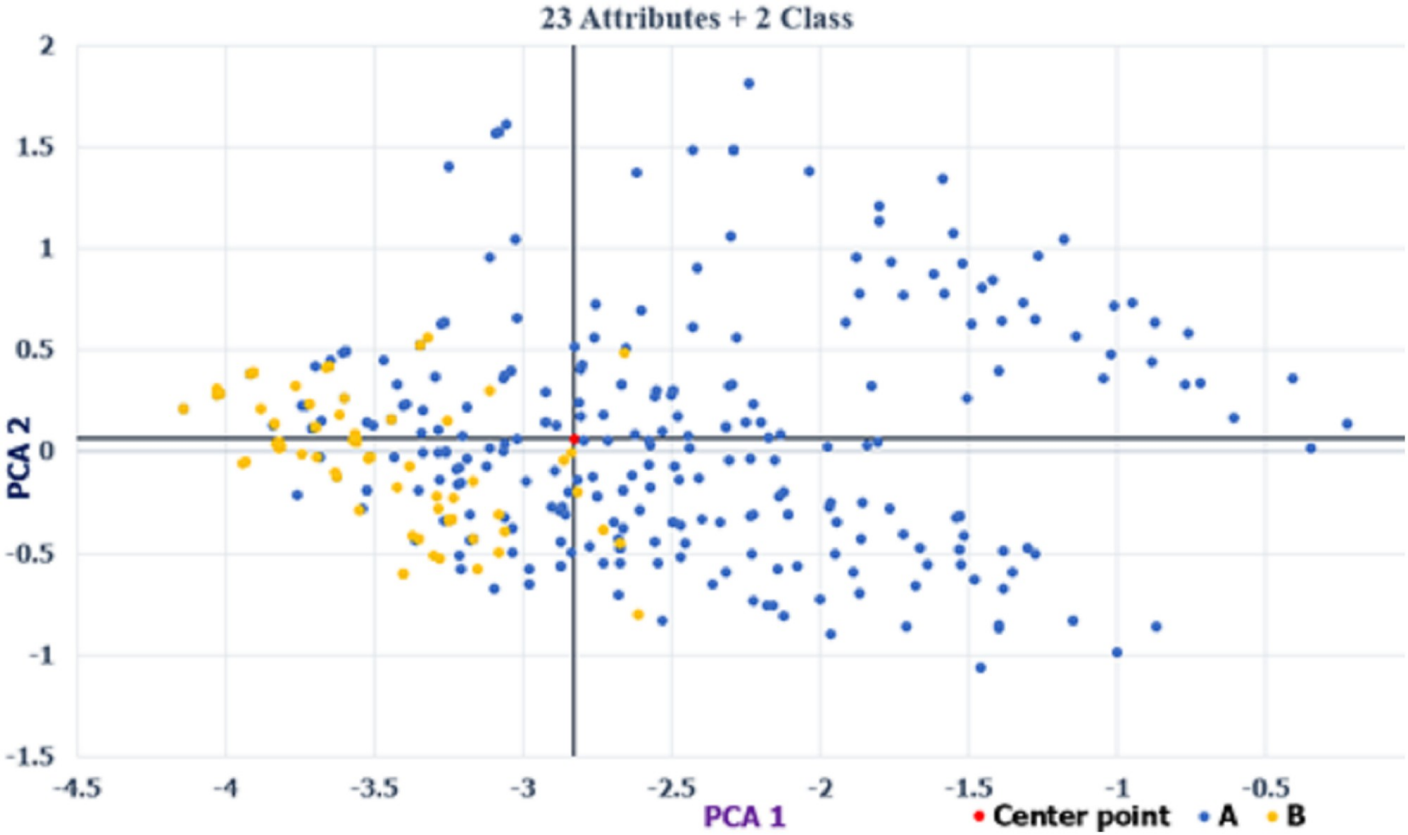
Scatter of PCA for two classes (23 attributes).

**Fig 4:**
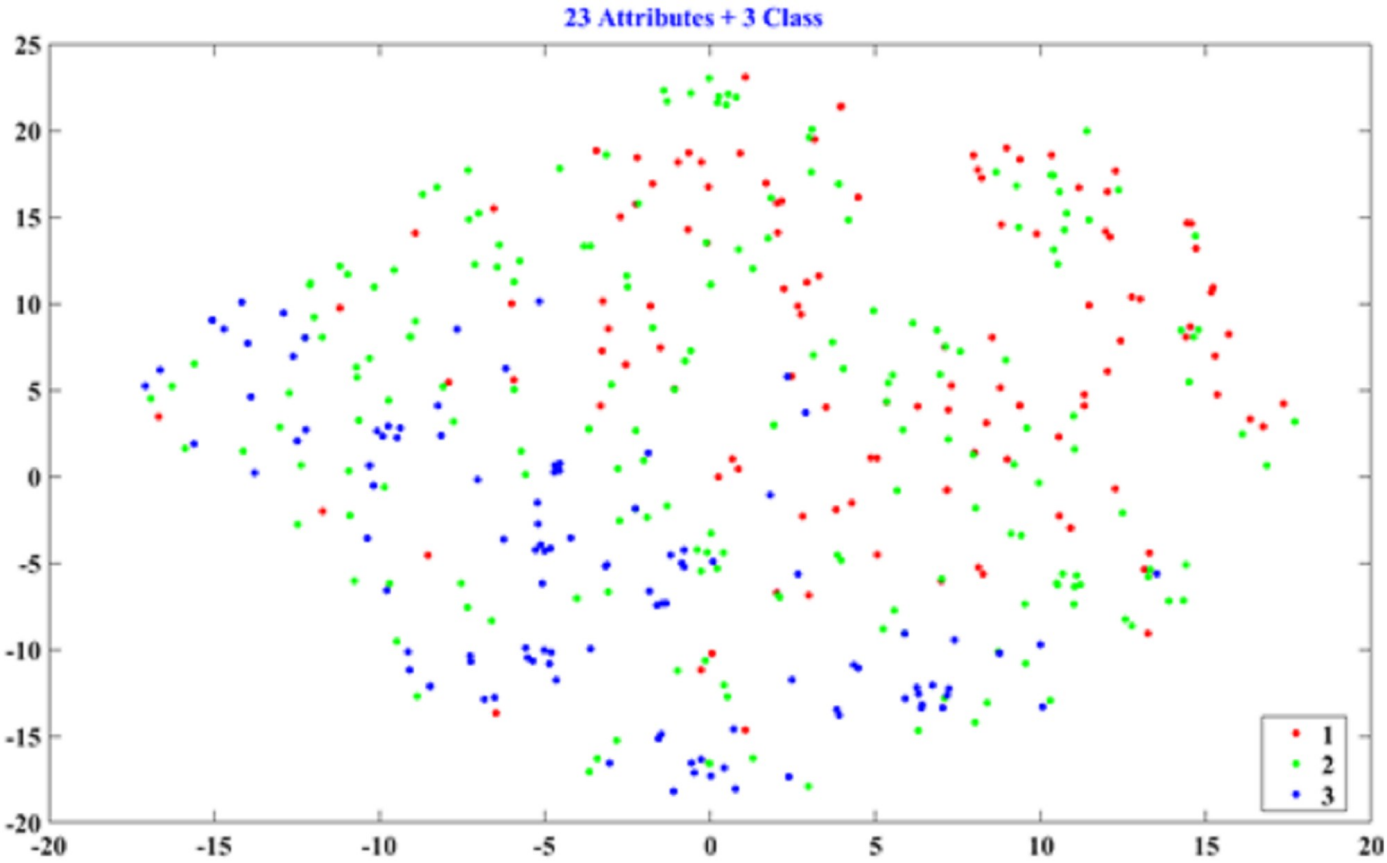
Scatter of t-SNE for three classes (23 attributes).

**Fig 5:**
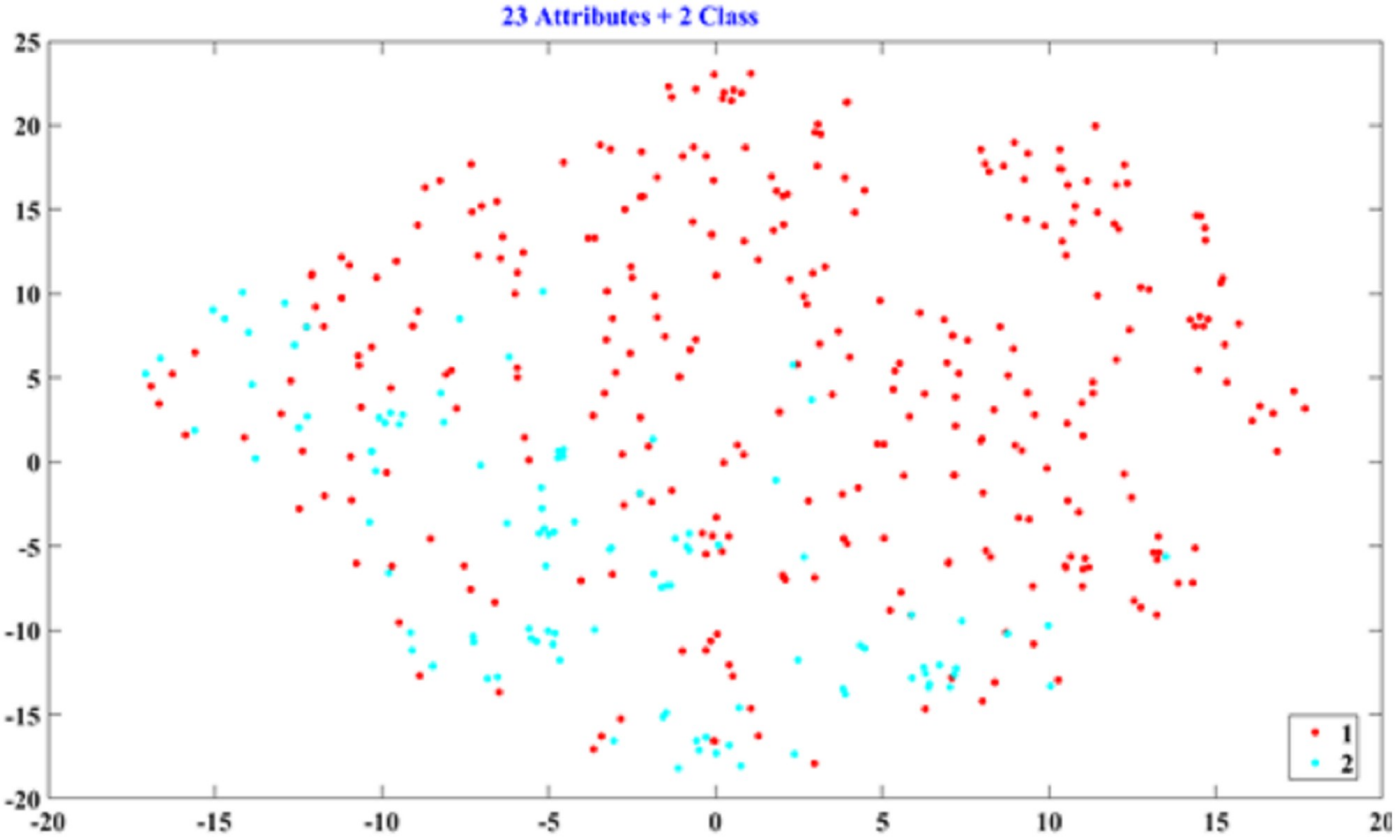
Scatter of t-SNE for two classes (23 attributes).

This section uses the patterns of PCA and t-SNE to determine which number of classes has a discriminating ability for hotel classification. We used the full attributes (23) for two and three classes to plot their PCA and t-SNE patterns. The PCA scatter diagrams are shown in Figs 2 and 3, and the t-SNE patterns are presented in Figs 4 and 5. As shown in Figs 2–5, the two-class patterns feature greater discriminating abilities than the three-class patterns. Therefore, this study used the two classes to generate the rules of hotel classification and the attribute performance matrix.

#### E. Accuracy

This study used tree C4.5 [35], Rough Set [31], Random Forest [42], Random Tree [49], and LibSVM [55] to classify the full and selected attributes data for the two and three hotel classes; the results were shown in Table 4. The accuracy of the two classes is higher than the accuracy of the three classes. As seen in Table 3, the three classes had selected eight important attributes, and the two classes had screened 10 important attributes. Then, we used the five classifiers to classify the data for the selected attributes. The results also show that the accuracy of the two classes was better than the accuracy of the three classes, as shown in Table 4. In addition, the rough set classifier has better accuracy than the other classifiers.

**Table 4:** The results of different classifiers.

Dataset	Tree	Rough Set	Random Forest	Random Tree	LibSVM
23 Attributes (3 classes)	59.56(6.95)	60.03(6.81)	**61.04**(6.90)	58.13(7.05)	56.10(6.42)
23 Attributes (2 classes)	81.21(5.50)	**89.48(0.64)**	83.86(5.24)	82.38(5.54)	81.96(5.81)
8 attributes (3 classes)	58.37(6.32)	56.78(6.51)	**59.33**(6.53)	58.72(6.37)	57.98(6.53)
10 attributes (2 classes)	81.95(5.54)	**84.81(5.36)**	83.39(5.58)	82.94(5.50)	83.16(5.50)

**Note:** each numeric cell denotes the average accuracy and the standard deviation in the bracket.

#### F. Attribute performance visualization

**Fig 6:**
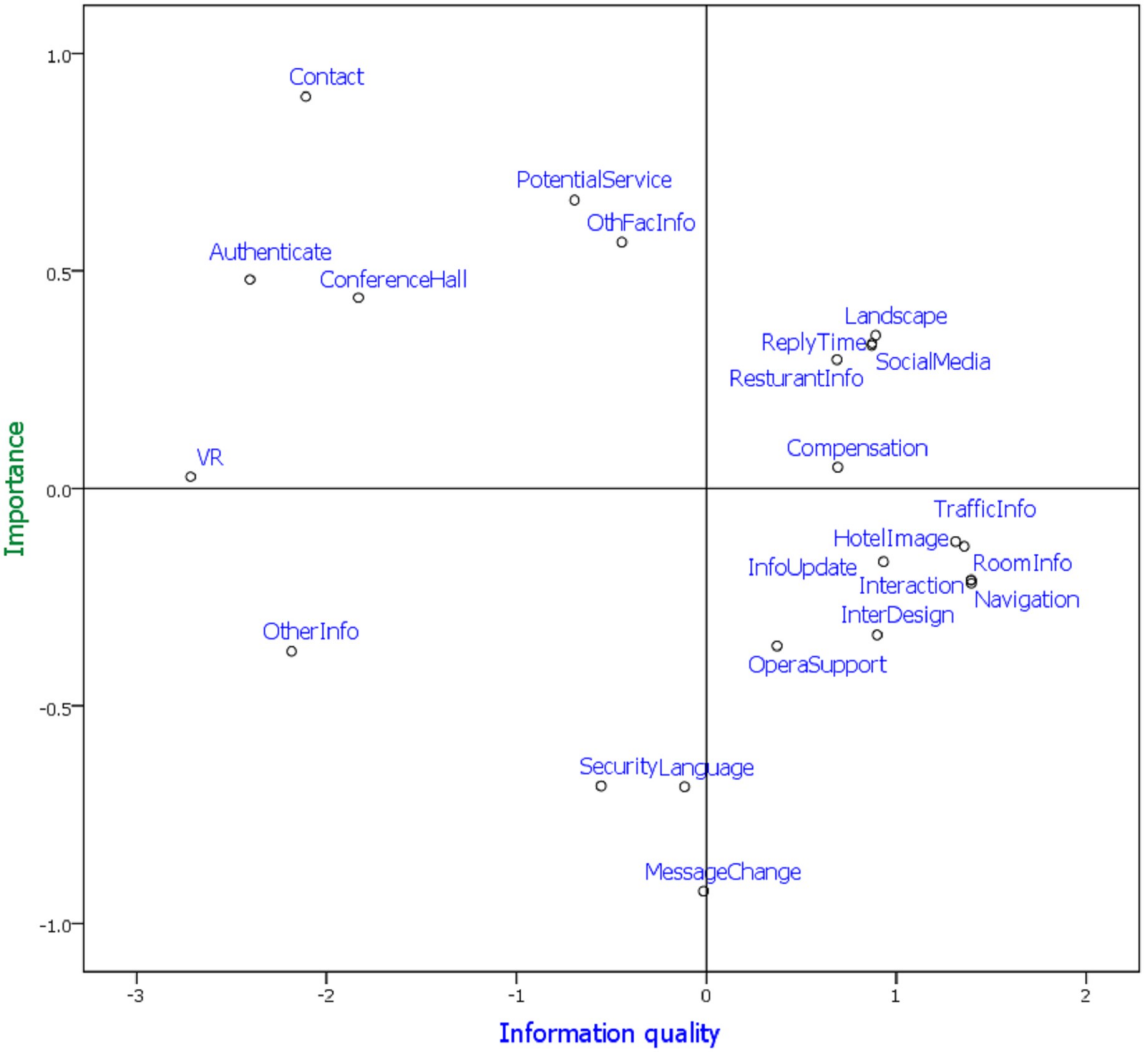
Attributes performance matrix (23Attributes+ 2Class, class A).

The ALSCAL method is used to determine the key attributes of each class; the highest accuracy is found in two classes with 23 attributes. This study applied the ALSCAL method to visualize attribute performance. The attribute-performance visualization of class A in Fig 6 is explained as follows.

1. Visible attributes: The visible information on the websites of one-star and two-star hotels includes room information, traffic information, interactive services, hotel image, information update, navigation, operational support, and interface design, as shown in the lower right section of Fig 6. Interactive services are essential for lower-star hotels, as they allow customers to quickly contact the hotel for customer service, while the hotel image gives customers a sense of the hotel.
2. Competitive advantages: As seen in the upper right section of Fig 6, the competitive advantage attributes of one-star and two-star hotels are landscape, reply time, restaurant information, social media, and compensation. Hotels with these attributes can easily attract travelers to book rooms.
3. Key focus attributes: If one-star and two-star hotels invest in these facilities (contact, authentication and cooperative symbols, conference hall, potential services, and information on other facilities), as shown in the upper left area of Fig 6, then they can achieve a competitive advantage.
4. Weak attributes: The weak attributes of the one-star and two-star hotels are other information, security, language, and message change, as shown in the lower-left section of Fig 6. If the one-star and two-star hotels want to attract travelers, they must improve these attributes.

**Fig 7:**
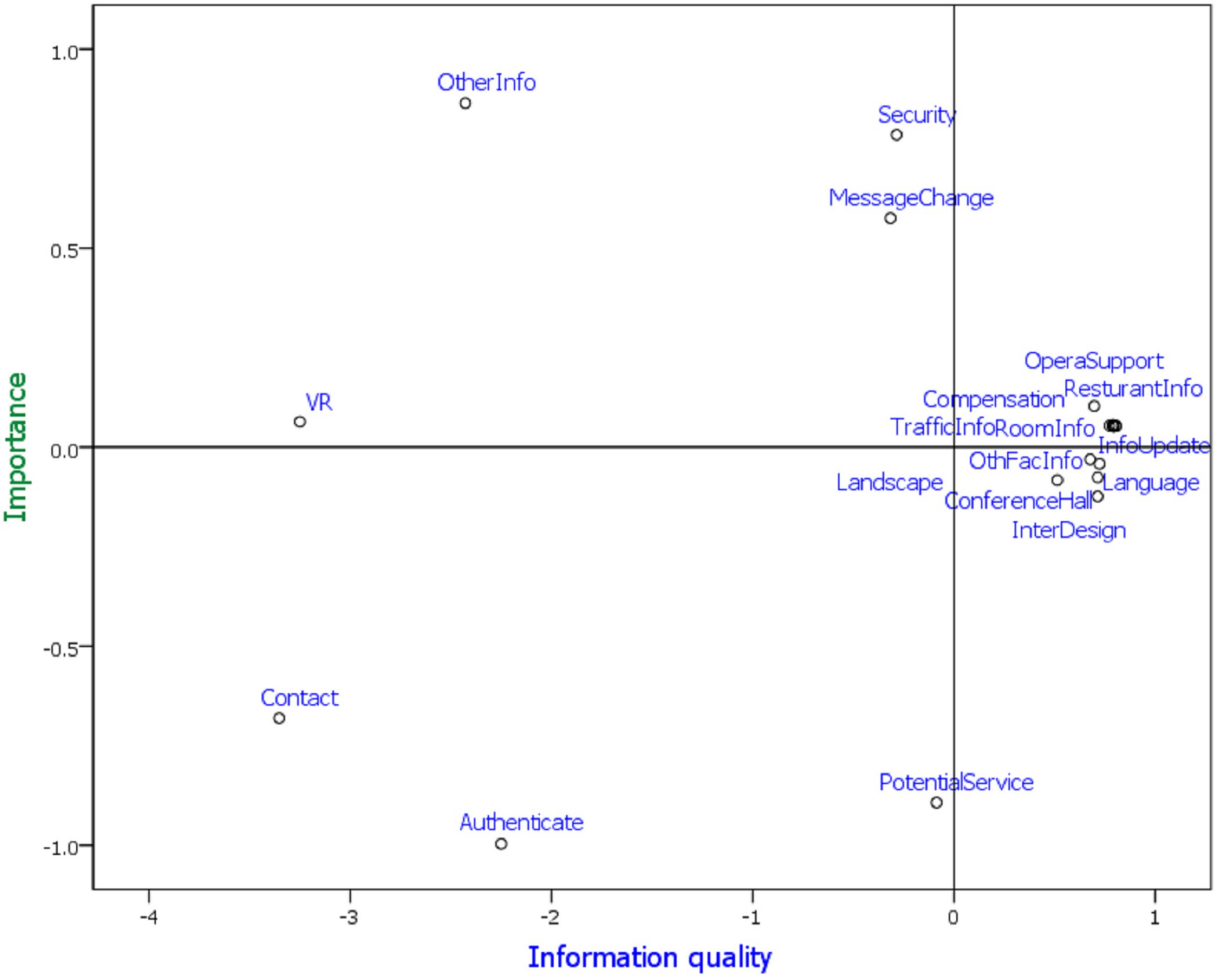
Attributes performance matrix (23Attributes+ 2Class, class B).

Similarly, the attribute-performance visualization of class B in Fig 7 is summarized as follows.

1. Visible attributes: The visible attributes of the three-five star hotels are landscape, language, interface design, conference hall, and information on other facilities. The three-five star hotels overstate their attributes. Hence, they must improve these attributes for customers to have a good perception of the hotel, especially in language.
2. Competitive advantages: The competitive advantage attributes of the three-five star hotels are operational support, compensation, restaurant information, traffic information, and room information. These attributes can easily attract travelers to three-five star hotels.
3. Key focus attributes: If the three-five star hotels improve their other information, security, message change, and VR, they can become advantageous attributes.
4. Weak attributes: The weak attributes of the three-five star hotels are contact, authentication, and potential service. The three-five star hotels must improve these attributes to attract travelers.

#### G. Generate the rules of the star-rated class

The rough set classifier is better than the other classifiers in its accuracy. Hence, this study used the rough set classifier to generate the rules of hotel classification. The accuracy of the three classes is not good; therefore, we only generated the rules for two classes with 23 Attributes, as shown in Table 5. The highest match rule of class A (one-star and two-star hotels) is 115, and the highest match rule of class B (three-star, four-star, and five-star hotels) is 13.

**Table 5:** The partial rules of hotel star rating (2 class).

Class	Rule	Match
**A**	(RoomInfo = Y)&(Navigation = Y)&(Interaction = Y)&(TrafficInfopInfo = Y)&(HotelImage = Y)&(VR = N)&(OtherInfo = N)&(ConferenceHall = N)&(OthFacilityInfo = N) = >(class = {A[115]})	115
**B**	(RoomInfo = Y)&(ResturantInfo = Y)&(Compensation = Y)&(InfoUpdate = Y)&(Navigation = Y)&(Interaction = Y)&(HotelImage = Y)&(TrafficInfopInfo = Y)&(SocialMedia = Y)&(ReplyTime = Y)&(Language = Y)&(ConferenceHall = Y)&(OthFacilityInfo = Y)&(InterDesign = Y)&(Landscape = Y)&(OperaSupport = Y)&(VR = N)&(OtherInfo = N)&(Authenticate = Y)&(MessageChange = Y)&(Security = Y) = >(class = {B[13]})	13

### 4.3 Discussions

After the analysis and experiment, we offer some discussions as follows.

#### A. Key attributes

Website quality is a multidimensional aspect that affects consumer attitudes and induces satisfaction [82], and it includes many attributes. Identifying the key attributes that influence the star-rated hotel allows consumers to differentiate between different competitors. Therefore, this study applies a rough set to find key attributes as follows.

1. The rough set can also find the core attributes by using an attributes reduction. Reduction refers to the deletion of one or more attributes in the original dataset. The remaining attributes can still achieve the classification accuracy of the original attribute. The deleted attributes are called unnecessary attributes, while the remaining attributes are necessary attributes. Here, the core attributes of the dataset are found by rough set reduction. We used a dataset of the highest accuracy (two classes with 23 Attributes) to execute the rough set reduction and the result is shown in Table 6.
2. The definition of the core attributes is the intersection of all reduction sets [32]. Then, we can delete the attributes of SocialMedia and ReplyTime. The two attributes are not core attributes (also called unnecessary attributes). The 15 retained attributes are the core attributes, as shown in Table 6.
3. We checked the collected dataset. All the values for the room, navigation, and interaction attributes are 1. This means that all 428 star-rated hotel websites have the same information quality. Based on data mining, the three attributes have no discriminating ability. Thus, we can directly delete them. The room, navigation, and interaction attributes are not listed in Table 6.
4. In the attribute selection of the two classes, as shown in Table 3, the website information of the star-rated hotels, especially international hotels, must consider the 10 key attributes to meet their customers’ needs. Many enterprises will rent conference halls from star-rated hotels as locations for activities or meetings, while the other facility information is of concern to customers. Moreover, star-rated hotels must disclose current information on their equipment, such as swimming pools, playrooms, and business centers.

**Table 6:** Reduct set of two classes with 23 attributes.

SC	Reducts
0.51	{ResturantInfo, ConferenceHall, OthFacilityInfo, Landscape, Compensation, Contact, InterDesign, OperaSupport, Language, VR, MessageChange, SocialMedia, Authenticate, Security, OtherInfo, PotentialService}
0.51	{ResturantInfo, ConferenceHall, OthFacilityInfo, Landscape, Compensation, Contact, InterDesign, OperaSupport, Language, VR, MessageChange, Authenticate, Security, ReplyTime, OtherInfo, PotentialService}
Core attributes	
ResturantInfo, ConferenceHall, OthFacilityInfo, Landscape, Compensation, Contact, InterDesign, OperaSupport, Language, VR, MessageChange, Authenticate, Security, OtherInfo, PotentialService	
Key attributes	
Conference Hall, OthFacInfo, Language, Compensation, Restaurant info., Social media, Reply time, Info update, VR, and Potential service	

Note: Stability Coefficient (SC) is a measure of similarity for class among all attributes; the higher value represents the higher relationship of similarity.

As mentioned above, we conclude that the key attributes are Conference Hall, OthFacInfo, Language, Compensation, Restaurant info., Social media, Reply time, Info update, VR, and Potential services, as shown in Table 6.

#### B. Data visualization

In the PCA and t-SNE patterns, the two-class patterns feature more discriminating abilities than the three-class patterns, as shown in Figs 2–5. However, for the data volumes used for data visualization, the t-SNE is superior to PCA. Because the explained variance of the first and second PCA scores is 22.75%, PCA cannot represent the patterns of all data. In addition, the t-SNE can project high-dimensional data onto a low-dimensional space for data visualization. In this study, we can directly determine the number of classes by their t-SNE pattern.

Hotel website information quality is a suitable method to differentiate competitors by evaluating the website quality of each hotel [64]. Users of hotel websites have different opinions on the overall quality of hotel websites with different star ratings. The importance of user-perceived website quality generally increases with the hotel’s rating. Therefore, the service quality of the hotel website should be consistent with its star rating. Based on the attribute performance visualization, the one-star and two-star hotels have competitive advantage attributes in their landscape, reply time, restaurant information, social media, and compensation. However, they must improve their other information, security, language, and message change to attract travelers. Furthermore, the three-five star hotels have competitive advantage attributes in their operational support, compensation, restaurant information, traffic information, and room information. However, they also have shortcomings in their contact, authentication, and potential service.

#### C. Classification rules

To simplify the rules of the star-rated hotels, this study used two classes with 10 key attributes to generate the tree.. The results show that the four critical attributes have 74.42% accuracy in generating the five rules. The highest match rule (225 hotels) for the one-star and two-star hotels is as follows:

If ConferenceHall is No, then Class = A.

In addition, the highest match rule of the three-star, four-star, and five-star hotels is:

If (ConferenceHall is Yes) and (Compensation is Yes) and (Language is Yes) and (OthFacInfo is Yes)

Then Class = B.

Roughly speaking, the collected data can be represented by the combined rules of the four important attributes (ConferenceHall, Compensation, Language, and OthFacInfo). In this way, we can easily discriminate the information quality of the website for star-rated hotels.

#### D. Practical and theoretical implications

This study employed two ANOVA analyses to show the higher star ratings hotels are better than the lower star ratings in terms of the important information quality of their websites in C of Section 4.2, and the information quality provided by the higher star hotels was more detailed than that offered by low-star hotels. According to agency theory [83], travelers face adverse selection problems or the existence of pre-contractual information asymmetry when choosing providers/hotels. This asymmetry of information increases in electronic environments [84]. According to signaling theory [85], star rating acts as a signal or cue of the overall quality and reputation of hotels; it diminishes uncertainty by helping consumers infer information about hotel characteristics. A hotel’s star classification is considered an important signal because higher classifications indicate better service attributes and quality hospitality. Based on signaling theory, we suggest that hotels use certain mechanisms or signals to help consumers infer the unobservable qualities of their products, services, and behavior, thereby reducing uncertainty. Such as enhancing website information quality reduces information processing or gap and helps consumers evaluate the quality of experiential hotel services [86].

## 5. Conclusion

This study proposed a novel methodology to explore the relationship between hotel star rating and website information quality; this novel methodology applied rough set theory (RST) and visualized tools (PCA and t-SNE patterns, and attribute performance visualization) to analyze the relationship between hotel star ratings and hotel information quality. The results showed that there were significant differences in information quality between hotels below two stars and above four stars. The information quality provided by the higher-star hotels was more detailed than that offered by low-star hotels. Lastly, the different star-rated hotels can be identified by a set of rules from the four critical attributes (ConferenceHall, Compensation, Language, and OthFacInfo). In this way, we can easily discriminate the information quality of the website for star-rated hotels.

Many foreign tourists are unable to access a hotel in person. Hence, the information services on the website are the only source of information that some tourists can rely on to choose the hotel they want to stay. This study also speculated that consumers would pay better attention to the available if hotel managers were to update their websites with new features, such as virtual reality. This higher information quality would attract many tourists. Virtual reality is a closer reality than general photos. Therefore, using virtual reality to browse hotel facilities could improve the consumer’s willingness to choose the hotel. Moreover, the hotel’s seal is less visible to consumers, especially for lower star hotels. Therefore, if the hotels display a government seal on their websites, customers would feel more at ease, thereby increasing the hotel’s reputation.

The data were collected from the Ministry of Transportation and Communications in 2019. The number of hotels may increase or decrease in the future. Hence, the results will be different. In addition, this study did not include homestays or non-star hotels; therefore, the results are not generalizable to non-star hotels. However, there are also plenty of homestays and non-star hotels across Taiwan; these data could be added to future research for analysis, likely producing different results. Therefore, we address the limitations of this study for future development as follows.

1. **Strengthening the number of attributes**
  This study is based on Hung’s website service quality with 23 attributes [64] to collect the websites of the 428 hotels data; future work could refer to more studies to collect more attributes, such as HWebSQ scale [87].
2. **Balancing the number of classes**
  The data collected are class imbalanced because the sample sizes for each class are not approximately equal. Therefore, this study attempted to merge the star-rated hotels into a balanced class but did not achieve this goal. Future work could be handled by undersampling, oversampling, and SMOTE [88] to address the limitation.
3. **Adding the number of samples**
  Only 453 star-rated hotels are collected. Therefore, we can expand the geographical area to increase the number of samples, such as large areas of China.

**Software and Code:** In this study, we use four software tools, which are introduced as follows.

Rough sets: this study applies RSES (Rough Set Exploration System, https://rseslib.mimuw.edu.pl/index.html) to implement all related results of rough sets. RSES is an open toolkit for analysis of table data, based on methods and algorithms coming from the area of Rough Sets.

t-SNE: this study uses t-SNE codes from the website: https://github.com/rahulvigneswaran/tsne-plotter. The open t-SNE codes are run in a commercial MATLAB platform.

PCA: this study applies a commercial IBM SPSS package v22 to show all figures.

ALSCAL: we use a commercial IBM SPSS package v22 to analyze the results.

Rseslib 3—Rough Set and Machine Learning Open Source in Java.
